# Supplementary material for: Droplet and fibril formation of the functional amyloid Orb2
Source: J Biol Chem. 2021 May 25;297(1):100804. doi: 10.1016/j.jbc.2021.100804 (PMC8294575; doi:10.1016/j.jbc.2021.100804)
Supplement: Figures S1–S6 [file mmc1.pdf]

Supplemental information for: Droplet and fibril formation of the functional amyloid Orb2

Kidist Ashami[a], Alexander S. Falk[a,1], Connor Hurd[a], Samridhi Garg[a,1], Silvia A. Cervantes[a], Anoop Rawat[a], Ansgar B. Siemer[a]

[a]*Department of Physiology and Neuroscience, Zilkha Neurogenetic Institute, Keck School of Medicine, University of Southern California, 1501 San Pablo Street, CA 90033, Los Angeles, USA*

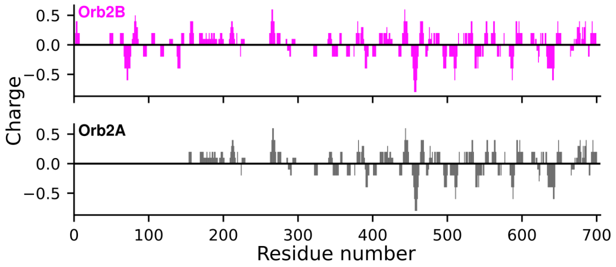

Figure S1: Charge density of Orb2B and Orb2A calculated using the 5 residue window showing that the charges are distributed relatively equal throughout the sequence.

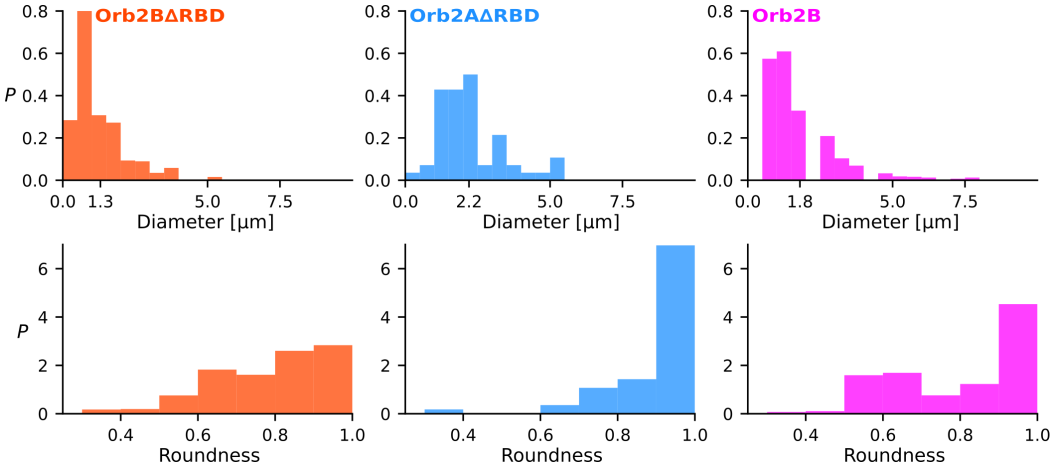

Figure S2: Orb2 droplets are generally between 0 and 5 µm in diameter and have a high degree of roundness. Diameter and roundness of Orb2 droplets were determined using the ImageJ BioVoxxel plugin. Histograms are presented as probability density P and the average diameter is indicated.

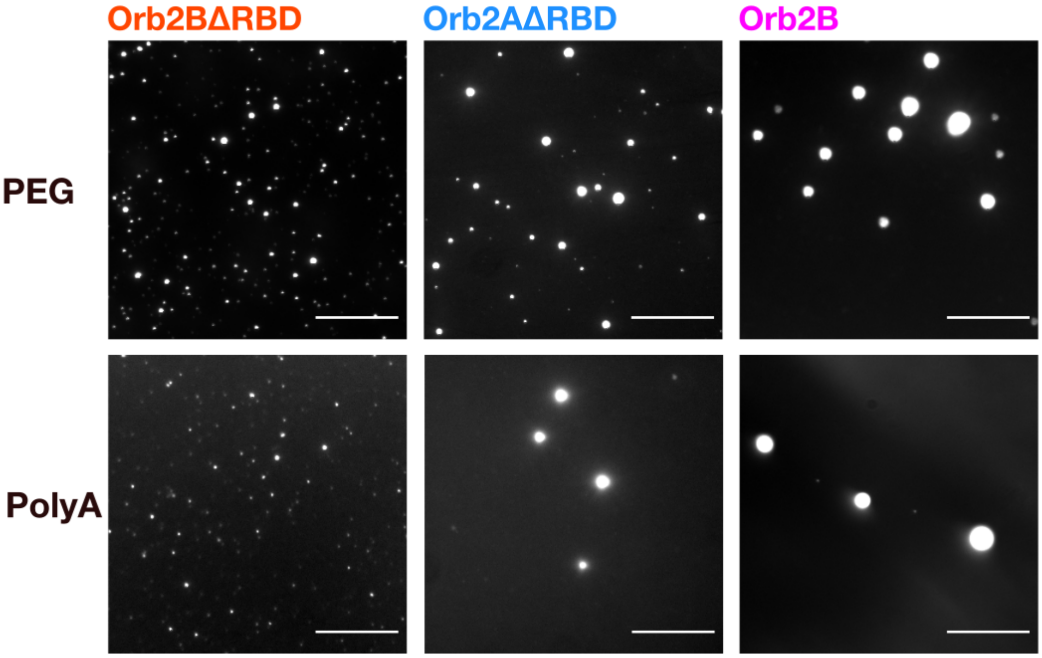

Figure S3: Orb2 also forms droplets in the presence of PEG and RNA. Fluorescence microscopy images of droplets formed by Oregon Green 488 labeled Orb2BΔRBD, Orb2AΔRBD, and Orb2B immediately after adding 10% v/v PEG 8000 or PolyA at a 1:10 PolyA:protein m/m ratio. Scale bars represent 20 μm.

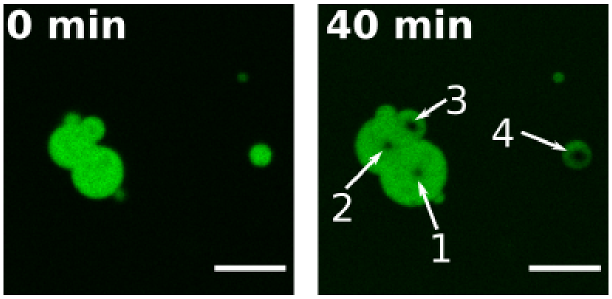

Figure S4: Orb2B shows neither FRAP recovery nor droplet fusion after 40 minutes. Merged Oregon Green 488 labeled Orb2B droplets before (0 min) and after 4 consecutive (indicated with numbers 1-4) 10 minute FRAP experiments (40 min). The fact that neither fluorescence recovery nor droplet fusion events could be observed indicates the relatively static nature of Orb2B inside these droplets. Scale bars represent 5 μm

.

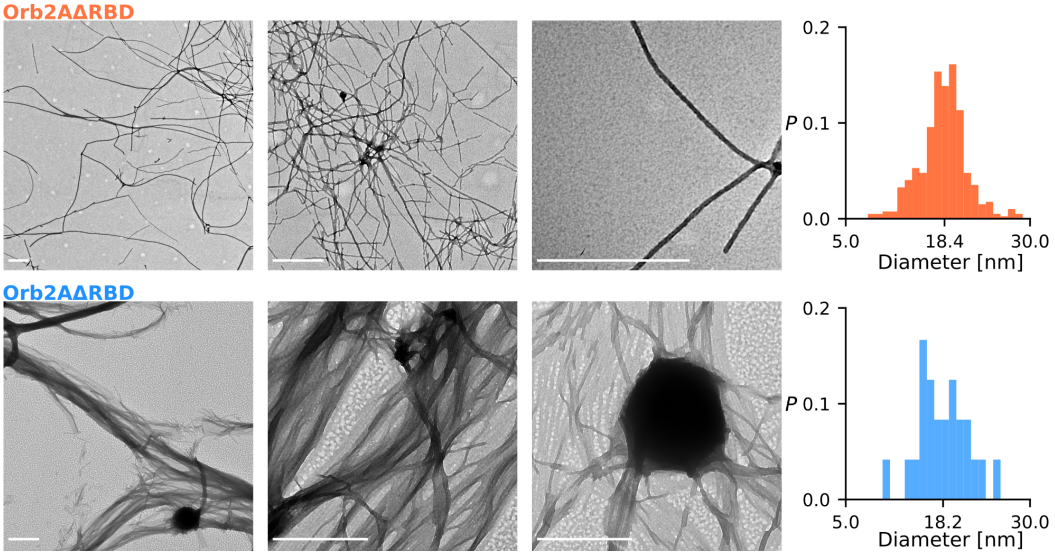

Figure S5: Orb2BΔRBD fibrils are relatively unbundled, whereas Orb2AΔRBD fibrils are highly bundeled. Both fibrils have a diameter of about 18 nm with no visible twist. Negative stained EM images of Orb2BΔRBD and Orb2AΔRBD fibrils 48 h after buffer exchange. Scale bars denote 500 nm. Fibril diameters were measured using the ImageJ FibrilJ plugin. Histograms are presented as probability density P and the average diameter is indicated.

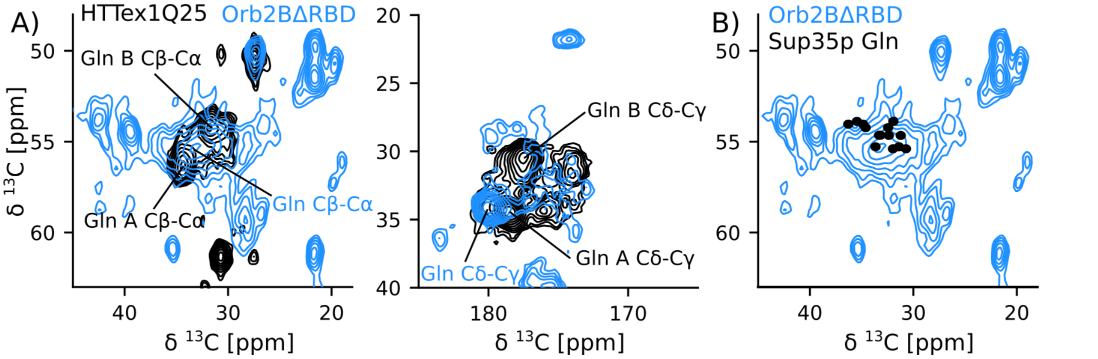

Figure S6: Comparison of Gln peaks in Orb2BΔRBD fibril spectra with HTT_{ex1} (Q25) and Sup35p data. A) Overlay of 2D ^{13}C-^{13}C spectra of Orb2BΔRBD shown in blue and HTT_{ex1} shown in black. Gln peaks are labeled in the same colors. Where HTT_{ex1} has two major Gln conformations Gln A and Gln B, Orb2BΔRBD has only one. The Gln peak of Orb2BΔRBD does not overlap well with either Gln A and Gln B but its Cδ-Cγ peak overlaps with the minor HTT_{ex1} Gln conformation Gln C. The HTT_{ex1} (Q25) spectrum was recorded using the 2D DARR experiment at 25 kHz MAS with a mixing time of 50 ms. Orb2BΔRBD spectrum shown in left panel is a 2D DREAM recorded at 30 kHz MAS, the right panel shows a 2D DARR recorded at 25 kHz MAS with a mixing time of 50 ms. B) Spectrum of Orb2BΔRBD fibrils with black circles indicating the Gln assignments in the core of Sup35p fibrils (BMRB access code 18407). Although not perfect, the Gln resonances of Sup35p are more similar to Orb2BΔRBD compared to HTT_{ex1}.
